# Enhancing Autonomy-Connectedness in Patients With Anxiety Disorders: A Pilot Randomized Controlled Trial

**DOI:** 10.3389/fpsyt.2019.00665

**Published:** 2019-09-12

**Authors:** Joyce Maas, Ton van Balkom, Marcel van Assen, Liesbeth Rutten, Daniella Janssen, Marietta van Mastrigt, Marrie Bekker

**Affiliations:** ^1^Department of Medical and Clinical Psychology, Tilburg University, Tilburg, Netherlands; ^2^Department of Psychiatry GGZ inGeest, Amsterdam, Netherlands; ^3^Multidisciplinary Specialist Center Centre for Eating Disorders, GGZ Oost-Brabant, Helmond, Netherlands; ^4^Department of Methodology and Statistics, Tilburg University, Tilburg, Netherlands; ^5^Department of Sociology, Utrecht University, Utrecht, Netherlands; ^6^Geriatric Psychiatry, Parnassia, The Hague, Netherlands; ^7^Department of Clinical Psychology, VU University, Amsterdam, Netherlands

**Keywords:** autonomy-connectedness, autonomy-enhancing treatment, transdiagnostic, anxiety, gender

## Abstract

Autonomy-enhancing treatment (AET) is a person-centered, gender-sensitive treatment, targeting transdiagnostic personal autonomy deficits. The current study was set up as a first pilot randomized controlled trial (RCT) to investigate the preliminary efficacy of AET. Earlier small non-controlled plots showed AET to be feasible and acceptable. In the current study (Trial Code 3513), patients receiving 15-session group-based AET (*N* = 43) were compared with those in a waitlist control condition (*N* = 40). Both the intention-to-treat and completers analyses suggested a larger decrease in agoraphobic symptoms in the experimental treatment than in the waitlist condition. In both analyses, effect sizes were small. The completers analyses showed additional beneficial effects in two of three autonomy-connectedness components, as well as psychoneuroticism, anxiety, and depression, which disappeared after correcting for multiple testing. AET may alleviate agoraphobic symptoms in a patient sample with severe anxiety. Future research, including more stringent inclusion criteria and follow-up assessment, is needed to further evaluate whether AET may serve as a promising alternative or addition to existing approaches.

**Clinical Trial Registration:** www.trialregister.nl, identifier NTR3513.

## Introduction

According to epidemiological studies, anxiety disorders are the most prevalent of all mental disorders: Lifetime prevalence estimates are as high as 28.8% (1). Cognitive behavioral therapy (CBT) is considered the first-choice treatment for anxiety disorders, and reported effect sizes are generally large (2–4). However, meta-analytic research shows that there is still significant need for improvement in the treatment of anxiety disorders [e.g., Ref. (3)]. For example, about one-third of patients do not respond to cognitive behavioral treatment, and approximately one-fifth drop out of treatment prematurely (5).

An important treatment challenge lies in the fact that anxiety disorders show high comorbidity (6) with other disorders such as depression and also with other anxiety disorders. Estimates of lifetime comorbidity rates for anxiety and depressive disorders are as high as 75% (7). Moreover, in case of symptom recurrence, the incidence of new anxiety disorders is common (8), suggesting an underlying deficit or vulnerability. This calls for transdiagnostic treatments targeting underlying mechanisms that may provide broader and more enduring treatment effects. Efforts have been made to develop and test cognitive-behavioral transdiagnostic treatments for emotional disorders [e.g., Ref. (9)]. However, less work has been done in the area of person-centered therapies. Person-centered treatments may be particularly beneficial in this regard, as they focus on self-pathology and more general factors underlying a diverse range of psychopathology. Examples are autonomy deficits, attachment issues (e.g., early maladaptive schemas), emotion regulation problems, identity problems, and interpersonal problems.

Autonomy-enhancing treatment (AET) is a person-centered approach used to treat transdiagnostic personal autonomy deficits. Originally, AET derived from feminist therapy and was only targeted for women with autonomy-related problems (e.g., difficulty setting boundaries and identity problems) (10). The more modern AET is targeted at both men and women and has recently been manualized (11). AET distinguishes itself from other person-centered treatments targeting self-pathology (e.g., schema-focused therapy, transference-focused therapy, and psychodynamic therapy) in being a relatively short treatment that aims to enhance the self and its self-steering capacity. It furthermore is a gender-sensitive treatment, explicitly focusing on different autonomy-connectedness needs for men and women.

More generally, AET is aimed at enhancing autonomy-connectedness. Autonomy-connectedness is informed by attachment theory (12, 13) and neo-analytical object-relation theory on the development of gender identity [e.g., Ref. (14)]. It entails the ability to identify and pursue one’s own wishes and needs while simultaneously engaging in satisfactory relationships (15, 16). That is, healthy psychological functioning does not only imply a strongly developed self, but also a strong connectedness to others. Autonomy-connectedness reflects a psychological state to be reached at the beginning of adulthood. Although autonomy stems from secure attachment experiences, and autonomy deficits are generated by insecure attachment (12, 13), not every insecurely attached individual will develop autonomy deficits. Bekker and Croon indeed found that although autonomy-connectedness and attachment were related, they were not confounded, which implies separate roles for autonomy-connectedness and attachment in the development of psychopathology. Conceptually, we would propose that insecure attachment styles predominantly reflect the styles of coping with insecure attachment experiences, whereas autonomy-connectedness deficits primarily reflect the resulting deficits in the self and its steering capacity.

More specifically, AET targets the three autonomy-connectedness components: self-awareness, sensitivity to others, and capacity for managing new situations (15, 16). The first two components reflect the capacity to be on one’s own as well as to be with others: Self-awareness is the capacity to be aware of one’s own opinions, wishes, and needs and the capacity to express these in social interactions, whereas sensitivity to others reflects sensitivity to the opinions, wishes, and needs of other people. It also includes empathy and the capacity and need for intimacy and separation. Capacity for managing new situations reflects how (un-)easy people feel in new situations and includes flexibility and an inclination to explore. Although this third component lies less at the core of autonomy, this drive to explore follows from secure attachment, which lies at the basis of healthy autonomous functioning.

Autonomy-connectedness deficits are characterized by low self-awareness and capacity for managing new situations and high sensitivity to others (15, 16). More specifically with regard to anxiety, these patients may not yet have a well-developed self, nor sufficient skills to identify their own needs (low self-awareness) as well as to enter new and potentially stressful situations (low capacity for managing new situations). Inversely, they may have learned to turn to others for help, and to ensure that their relationship to these others remains intact, by excessively monitoring these others’ needs (high sensitivity to others).

Deficits in autonomy-connectedness are hypothesized to be an important underlying vulnerability factor for developing mental health problems. Indeed, clinical research indicates that patients with anxiety disorders consistently score lower on autonomy (-connectedness) than do controls, with autonomy significantly predicting anxiety symptoms [e.g., Refs. (17–19)]. Autonomy deficits have also been associated with other various types of psychopathology such as depression [e.g., Refs. (20–23)], eating disorders (24, 25), aggression and personality disorders [e.g., Refs. (26, 27)], and work stress (28). Recently, Maas et al. (29) demonstrated that stressful life events make individuals more vulnerable for psychopathology, particularly in the presence of low autonomy-connectedness (29). Moreover, research suggests that autonomy-connectedness is relatively independent from personality factors (30) and only moderately associated with attachment styles (20).

The goal of AET is to strengthen self-awareness, regulate sensitivity to others, and enhance capacity for managing new situations. AET focuses on personal autonomy-related goals and addresses autonomy-connectedness with respect to multiple relevant life domains. Although AET is offered at many mental health care institutions, there is a lack of controlled treatment studies. So far, only two (unpublished) small uncontrolled pilot studies evaluated AET with the main aim to further develop and gain experience with the intervention. These pilots showed AET to be feasible and acceptable.

The present experimental study is the first to investigate the effects of group-based AET compared with a waitlist control condition in a large sample of outpatients with mixed anxiety disorders. We hypothesized that AET would increase autonomy-connectedness and quality of life and reduce anxiety-related and comorbid depressive symptoms, relative to the waitlist control condition.

## Materials and Methods

### Participants and Procedure

This parallel group multicenter pilot randomized controlled trial (Dutch Trial Register; Trial Code 3513) aimed to examine the relative efficacy of 15 sessions of AET compared with a waitlist control condition in a sample of patients with anxiety disorders. Participants were included between September 2012 and March 2014. The trial was predetermined to run during this period. The study was conducted at three mental health-care institutions in the Netherlands. The trial was approved by the medical ethics committee of the VU-University Medical Center and was carried out in compliance with the Code of Ethics of the World Medical Association (Declaration of Helsinki).

Participants had to be over 18 years of age and had to meet the *Diagnostic and Statistical Manual of Mental Disorders*, 5th Edition (DSM 5) criteria for one of the anxiety disorders as their main diagnosis. Patients were excluded if they suffered from a current psychotic episode, substance abuse disorder, acute bereavement, mental retardation, or suicidal thoughts or actions or when their Dutch language skills were insufficient. Patients were not allowed to receive additional treatment during the study, and patients’ psychotropic medication had to be unchanged for the past and coming 3 months. If additional treatment or a medication change was needed, the patient was excluded from the study (but was allowed to finish all treatment sessions if treatment had already started).

All possible eligible participants (*N* = 161) received written information on the study and provided consent to be contacted by the research assistant approximately a week later. If patients were interested to participate, they signed an informed consent. Patients were ensured that their choice not to participate (or to drop out during the study) would not negatively affect their treatment. Patients who decided to not participate received care as usual. In total, 107 provided written informed consent (66% consent rate).

**Figure 1:**
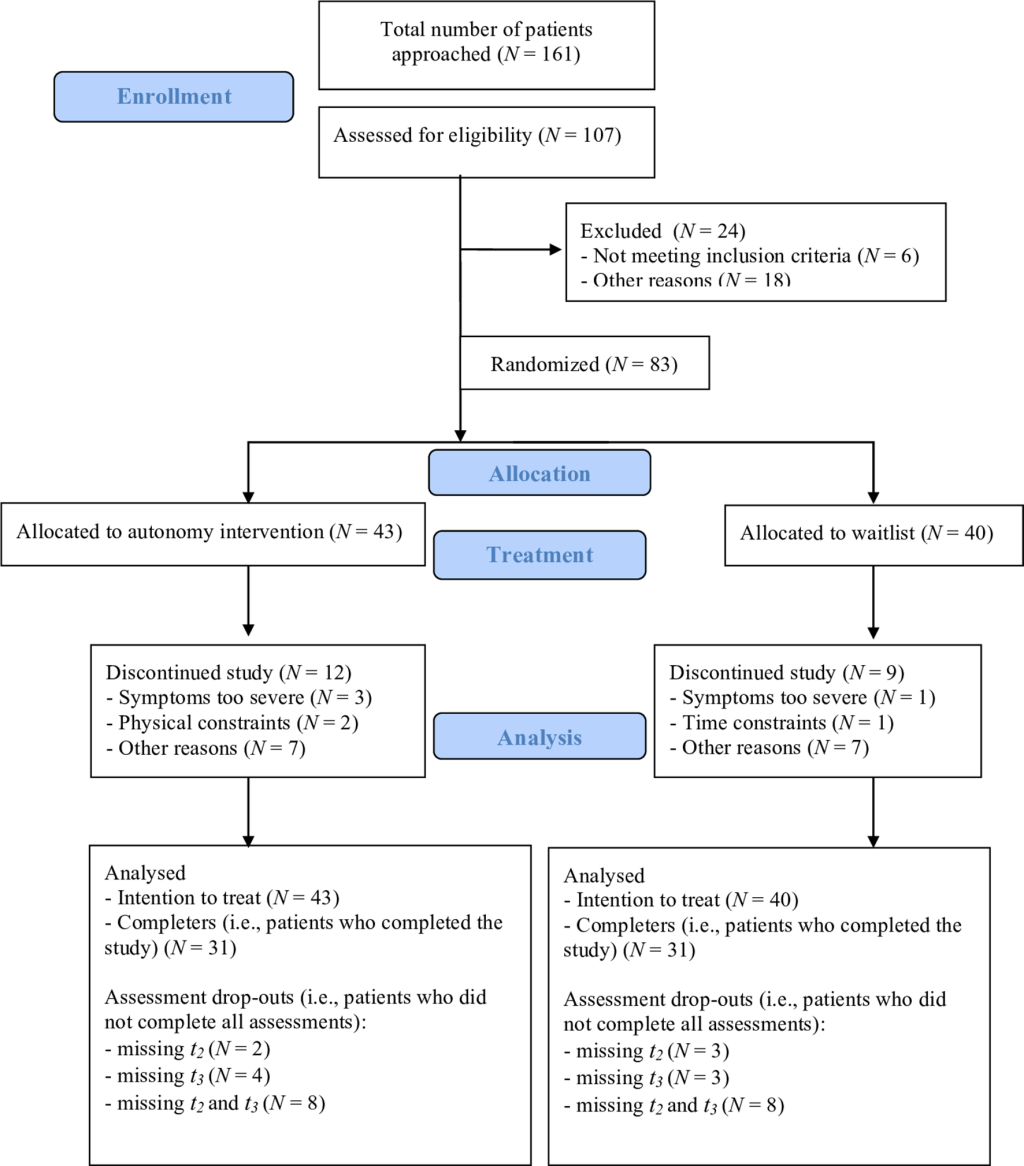
Patient flowchart.

Trained research assistants then screened patients who consented with both an intake interview and the MINI-International Neuropsychiatric Interview (31), as well as with a short screening checklist with the inclusion and exclusion criteria. After screening and the MINI interview, 83 patients were deemed eligible to participate. After completing the baseline assessment, these patients were randomized to either the waitlist or to AET. Randomization was accomplished by a methodologist not involved in the study who prepared a (random) list with patient numbers allocating patients to either of the two conditions. Four members of the data management team had access to this list and executed the randomization of the patients. Forty-three patients were allocated to the group AET condition, and 40 were allocated to the waiting-list condition (for power considerations, see the Data Analysis paragraph of the Methods section). **Figure 1** displays the flow diagram of patient inclusion, exclusion, and (reasons for) dropout of 24 participants. **Table 1** shows the sample’s clinical diagnoses.

**Table 1:** Patients’ baseline characteristics: clinical diagnosis.

Diagnosis	Percentage
Agoraphobia	48.2
Social anxiety	41.0
Generalized anxiety disorder	39.8
Panic disorder	32.5
Depressive disorder	31.3
Dysthymic disorder	15.7
Post-traumatic stress disorder	8.4
Obsessive compulsive disorder	1.2
Hypomanic episode	1.2
Bulimia	1.2

This table represents main diagnoses and comorbid diagnoses obtained with the MINI-International Neuropsychiatric Interview, which explains the high percentages that add up to more than 100%. Furthermore, all disorders are presented separately. For example, although some patients were diagnosed with panic disorder with agoraphobia, the table presents these diagnoses as separate disorders.

### Treatment

AET involved 15 weekly group meetings of 2 h each and focused on optimizing the three autonomy-connectedness components. The treatment was based on a manual with a session-by-session script (11). A group format allows patients to exchange their experiences and engage in therapeutic group exercises (e.g., distance-proximity exercises for learning to set boundaries). As autonomy-connectedness as a concept involves connectedness to others, a group format is preferred, although AET can also be delivered individually.

AET is gender sensitive and acknowledges that adult identity and autonomy develop differently in boys than in girls, because of their different relation with their primary attachment figure, usually their mother [e.g., Ref. (14)]. For many women, AET therapy goals often appear to include acknowledging other people’s needs and wishes but getting more in contact with those of their own and learning to make decisions more congruent with these own needs and wishes. For men, due to masculinity norms, AET therapy goals often include becoming aware of their vulnerability and dependency needs and expressing these in social interactions.

Sessions include two parts, separated by a break. During the first part, therapists deliver theme-based information. Themes that are discussed during the meetings are the following: “survival strategies” (i.e., strategies acquired in the past and at that time useful for facing harsh circumstances but in actual life maladaptive for healthy functioning); learning history; parent–child relationship and role models; the development, nature, and adaptiveness of schemas; setting boundaries; communication; body perception and sexuality; emotions and cognitions; friendship and relationships; saying goodbye; and relapse prevention. Every session, one patient is appointed as the chair, which further boosts patients’ autonomy-connectedness. Therapists have to make sure that every patient is chairing at least once and that patients’ personal autonomy-related goals are core to the discussions.

Two therapists guided the groups consisting of 6 to 10 participants each. In total, nine therapists were involved in the study. One of the group’s therapists was always a certified psychologist or a psychotherapist, assisted by another certified psychologist or a psychiatric nurse. All therapists had ample experience treating anxiety disorders. Two of the therapists, who also wrote the treatment protocol, trained and regularly supervised the remaining therapists. Fidelity was monitored by these supervisions and random checks of audio recordings of sessions. All sessions were recorded, and 20% of sessions were randomly selected and rated by the first author and an independent rater (a research assistant; a clinical psychology graduate student who was familiar with AET). The raters rated adherence to the treatment protocol’s components from 1 (missing) to 10 (perfect; all the material was discussed exactly as presented in the treatment protocol). More specifically, sessions typically consist of the following components, which were all rated for adherence: an awareness-increasing exercise; a round where each patient discusses to what extent and in what ways (s)he achieved his/her personal and autonomy-related goals during the past week; discussion of the weekly theme including an exercise; evaluation of the exercise and other issues patients would like to discuss; evaluation of the chair; and homework for the upcoming week. The average rating was 8.5. Agreement between raters was moderate (Cohen’s κ = .474).

### Materials

Questionnaires were completed at home (online or *via* paper and pencil) three times: at baseline (*t*_1_), after 7 weeks (*t*_2_; mid-treatment), and after 15 weeks (*t*_3_; post-treatment). When participants in the experimental treatment condition had to wait longer than 6 weeks before their group AET started, the baseline assessment was completed again directly before the start of treatment. Participants in the waitlist condition did not receive treatment and started with group AET after 15 weeks.

*Autonomy-connectedness* was assessed with the Autonomy-Connectedness Scale (ACS-30) (16), which consists of 30 items and three subscales: Self-awareness, Sensitivity to others, and the Capacity for managing new situations. All items are measured on a 5-point Likert scale, running from 1 (disagree) to 5 (agree). In the present study, Cronbach’s *alphas* for the subscales of the ACS were good:.78 for Self-awareness,.78 for Sensitivity to others, and.79 for Capacity for managing new situations.

*General psychopathology, Anxiety, and Agoraphobia* were measured with the Symptom Checklist-90 (SCL-90) (32) [Dutch translation, Ref. (33)]. The SCL consists of 90 items measured on a 5-point Likert scale ranging from 0 (not at all) to 5 (very much). There are nine subscales, known as Agoraphobic complaints, Anxiety, Depression, Somatic complaints, Insufficiency of thinking and acting, Interpersonal sensitivity, Hostility, and Sleeping problems. In the present paper, we used the total score as well as the Anxiety and Agoraphobia subscales. Reliability for the total score and the Anxiety subscale was excellent (Cronbach *alphas* of .97 and .87, respectively) and good for the Agoraphobia subscale (Cronbach *alpha* = .90).

*Agoraphobic complaints* were assessed with the Fear Questionnaire (FQ) (34). The questionnaire consists of 21 items with 15 agoraphobic fear-related items assessing situations patients avoid. The remaining items assess the patients’ most important fear, and other anxiety-related and depression-related information. In the present study, only the 15 agoraphobic fear-related items were used. These 15 items are measured on an 8-point scale with 0 meaning “I don’t avoid,” 4 meaning “I definitely avoid,” and 8 meaning “I always avoid.” Cronbach’s *alpha* of the FQ in the present study was.87, which reflects good reliability.

*Depressive symptoms* were assessed with the Beck Depression Inventory II (BDI-II) (35). The questionnaire consists of 21 items that are measured on a 4-point scale ranging from 0 (not feeling …) to 3 (feeling so … that I can not endure it). Cronbach’s *alpha* in the present study was excellent with a value of .92.

*Quality of life* was assessed with the WHO-QOL BREF (36), which consists of 26 items including four Quality of life domains: physical health, psychological health, social relationships, and environment. Additionally, two items examine general Quality of life. All items are rated on a 5-point Likert scale with 0 meaning “not at all,” “never,” “very poor,” or “very dissatisfied” and 4 meaning “completely,” “always,” “very good,” or “very satisfied.” In the current study, we used the total score, which had a Cronbach’s *alpha* of.93, which reflects excellent reliability.

Due to the small sample and focus of the present paper, only those measures directly relevant to the questions under investigation were included (see website of Dutch Trial Register Trial Code 3513 for the full list of measures).

### Data Analyses

A priori sample size calculation was performed with G*Power 3.1.3 (37). Assuming 15% dropouts and a significance level of.05, a total sample size of 76 was required for obtaining a power of.8 to detect a large effect size (*f* = 0.40) using a multivariate analysis of variance (MANOVA) repeated measures (two groups, three measurements). We used MANOVA for our power analyses instead of multilevel power analysis software, because MANOVA also accurately takes clustering of observations into account but does not require making sophisticated choices or guesses (which are required in multilevel power analysis). We included 83 patients of whom 62 completed treatment, resulting in a power of.79 to detect *f* = 0.4.

We used multilevel analyses to analyze the data, because, in contrast to the pairwise deletion procedure applied by repeated-measures ANOVAs, missingness can be handled well using a multilevel analysis (38). While repeated-measures analyses provide means and standard deviations for all time-points (using the pairwise deletion procedure), these are not directly available in the multilevel output. In contrast, multilevel analyses estimate condition “means” based on the complete model. In the present paper, we present the observed means and standard deviations for the baseline assessment (*t*_1_; **Table 3**) and the main and interaction effects estimated by multilevel analysis to evaluate effects over time and between groups (**Table 4**). We abstain from presenting the observed means at *t*_2_ and *t*_3_ because they are affected by missing data (e.g., those with less well-being dropped out at later points, biasing means at these time points), whereas means and effects predicted by multilevel are not.

More specifically, data were analyzed with generalized mixed models using IBM SPSS version 23. In order to increase statistical power, we examined if we could simplify the model (i.e., reducing the number of parameters). We first examined the covariance structure of the dependent variables. Based on the likelihood ratio test comparing the unstructured covariance matrix (six parameters) with the compound symmetry matrix (two parameters, corresponding to the random intercept model), we concluded that the random intercept model fitted the covariance structure of the dependent variables well. Second, we examined if we could simplify the fixed effects part of the model. Because the model with a Group × *t*_23_ interaction, which assumes equal interaction effects of Group with the second and third measurement dummies (*t*_2_ and *t*_3_), never fitted significantly worse than the model with both the Group × *t*_2_ and Group × *t*_3_ interactions, we only report results of Group × *t*_23_ interactions. Finally, because the main effects of *t*_2_ and *t*_3_ were similar (with the exception of the completers analyses of the ACS-30 Capacity for managing new situations subscale), we report the effects of *t*_23_, which represents the Time effect averaged across the last two measurements. The simplifications of the fixed effect model reduced model complexity with two parameters. Consequently, our main analyses are based on model *Y* *_it_* = β_0_ + β_1_ Group + β_2_ *t*_23_ + β_3_ Group × *t*_23_, with the intercept corresponding to the control group at *t* = 1, and Group and *t*_23_ corresponding to dummies for the treatment group and *t* > 1, respectively. The fact that main and interaction effects were similar for *t*_2_ and *t*_3_ implies no (statistically significant) changes were observed from *t*_2_ to *t*_3_.

In all analyses, a significance level of .05 was used. To assess clinical relevance of our findings, effect sizes were calculated by dividing the corresponding Time × Group effect by the square root of the mean squares within (39), which was obtained by running a one-way ANOVA on *t*_1_. We interpreted these similar to standardized mean difference Cohen’s *d*, with .2, .5, and.8 representing small, medium, large effect sizes, respectively. For every dependent variable separately, the following analyses were conducted. We first checked for an interaction of Time × Group, using one-tailed tests. A significant interaction was followed up by simple effects (one-tailed) to examine if the improvement was stronger for AET than for the waiting list condition. All analyses were conducted for both the full group (*N* = 83) and completers (*N* = 62). We defined completers as participants who remained in the study until the last assessment (treatment completers). Patients who completed all questionnaires but dropped out of the study prematurely (assessment completers) were not considered completers but were considered dropouts.

## Results

### Clinical Characteristics

The experimental treatment condition (*N* = 43) did not differ significantly from the waitlist condition (*N* = 40) with regard to sociodemographic variables and clinical characteristics (**Table 2**). There was also no difference across conditions in dropout rate, *t*(29) = 0.75, *p* = .46. **Table 3** displays descriptives for the dependent variables at *t*_1_ for the experimental treatment condition versus the waitlist condition and for the dropouts versus the completers. Dropouts scored significantly higher on the SCL Agoraphobia subscale and lower on Sensitivity to others and Quality of life than completers. The different treatment centers were also compared, but outcomes did not differ significantly between the treatment centers (range adjusted *r* squared: −.014 to.051). This factor was, therefore, not included in the multilevel analyses. Patients’ symptoms were severe. On average, the sample showed very low autonomy-connectedness scores (indicated by low Self-awareness and Capacity for managing new situations and high Sensitivity to others; Bekker and van Assen (16). Also, SCL scores were above mean standard scores for psychiatric patients (33). With a cut-off of 20, the BDI-II scores also indicate the presence of depressive symptoms (35). Most patients (73.2%) received previous treatment: 22 patients received one previous treatment, 13 received two, 24 received three, and 1 received four previous treatments.

**Table 2:** Patients’ baseline characteristics: sociodemographic and clinical variables.

	Experimental treatment (*N* = 43)	Waitlist (*N* = 40)	*t*/χ^2^
Age (mean, *SD*) Gender (*N*) Female	51.49 (16.07) 36	54.82 (13.79) 33	.84, *p* = .40 .01, *p* = .91
Male	7	6	
Nationality (*N*) Dutch	36	30	.60, *p* = .44
Other	7	9	
Level of education (*N*) Low	9	7	.27, *p* = .88
Middle	17	18	
High	13	12	
Previous treatment (*N*) Yes	33	27	.34, *p* = .56
No	10	11	
Psychopharmaca (*N*) Yes	20	21	.84, *p* = .36
No	23	16	

**Table 3:** Patients’ baseline characteristics: observed means (SD) at t_1_.

Questionnaire	Experimental treatment	Waitlist	*t*(df); *p*	Completers	Dropouts	*t*(df); *p*
ACS-30						
SA	20.83 (5.80)	19.68 (6.76)	−0.83(80);.41	19.77 (6.38)	21.80 (5.82)	−1.26(80);.21
SO	65.81 (10.68)	65.10 (10.11)	−0.31(80);.76	66.77 (10.19)	61.40 (9.99)	2.06(80);.04
CMNS	14.33 (5.63)	13.54 (6.40)	−0.59(80);.56	13.44 (6.03)	15.43 (5.75)	−1.32(80);.19
SCL-90						
Total	190.47 (51.17)	198.32 (57.47)	0.61(69);.55	193.23 (56.43)	200.00 (46.04)	−0.42(69);.68
Anxiety	24.62 (8.23)	25.13 (7.23)	0.29(77);.77	24.28 (7.13)	26.89 (9.37)	−1.27(77);.21
Agoraphobia	13.88 (7.08)	16.36 (7.62)	1.52(79);.13	14.00 (7.05)	18.58 (7.63)	−2.43(79);.02
FQ	33.63 (20.20)	38.97 (26.02)	1.02(76);.31	34.80 (22.69)	40.68 (24.93)	−0.96(76);.34
BDI-II	22.13 (12.43)	21.95 (11.01)	−0.07(75);.95	20.83 (11.54)	25.74 (11.60)	−1.61(75);.11
QOL	81.61 (15.73)	78.17 (15.45)	−0.97(72);.35	82.00 (15.61)	72.44 (13.42)	2.23(72);.03

ACS-30, Autonomy-Connectedness Scale-30; SA, Self-awareness; SO, Sensitivity to others; CMNS, Capacity for managing new situations; SCL-90, Symptom Checklist-90; FQ, Fear Questionnaire; BDI, Beck Depression Inventory; QOL, Quality of Life.

### Main Analyses

**Table 4** presents the full details with regard to the analyses. Most important with regard to our hypothesis is the Time × Group interaction in the first column. In case of a significant interaction, the Time effect is presented separately for both groups in the third column (simple effects analyses). If the Time × Group was not significant, main effects for Group and Time are presented in the second and third columns, respectively.

**Table 4:** Overview of the mixed model analyses for the intention-to-treat (ITT) and completers analyses (CA).

Measure	Analysis	Time × Group B(SE), p	Effect size (95% CI)	Group *B(SE), p*	Time *B(SE), p*
ACS-30					
SA	ITT	0.17 (0.96), *p* = .43	0.03 (−0.28, 0.33)	1.02 (1.28), *p* = .21	*t*_23_ = 1.03 (0.48), *p* = .02
	COMP	0.47 (1.04), *p* = .32	0.07 (−0.25, 0.40)	1.74 (1.47), *p* = .12	*t*_23_ = 1.42 (0.52), *p* = .004
SO	ITT	−2.20 (1.34), *p* = .052	0.21 (−0.04, 0.47)	−0.25 (2.11), *p* = .45	*t*_23_ = 0.063 (0.68), *p* = .46
	COMP	−2.45 (1.44), *p* = .046	0.23 (−0.04, 0.50)		Waitlist: *t*_23_ = 0.92 (0.94), *p* = .17 Treatment: *t*_23_ = −1.53 (1.10), *p* = .09
CMNS	ITT	0.82 (0.78), *p* = .15	0.14 (−0.12, 0.39)	1.36 (1.15), *p* = .12	*t*_23_ = 0.26 (0.40), *p* = .26
	COMP	1.48 (0.81), *p* = .04	0.24 (−0.02, 0.50)		^†^Waitlist: *t*_2_ = −0.79 (0.68), *p* = .12 *t*_3_ = 0.43 (0.67), *p* = .26 Treatment: *t*_2_ = 0.92 (0.65), *p* = .08 *t*_3_ = 1.69 (0.66), *p* = .01
SCL					
Total	ITT	−12.31 (8.37), *p* = .07	0.23 (−0.08, 0.53)	−16.04 (11.95), *p* = .09	*t*_23_ = −14.39 (4.25), *p* < .001
	COMP	−15.64 (8.71), *p* = .04	0.28 (−0.03, 0.58)		Waitlist: *t*_23_ = −4.15 (5.73), *p* = .24 Treatment: *t*_23_ = −20.75 (6.67), *p* = .002
Anxiety	ITT	−2.14 (1.30), *p* = .051	0.28 (−0.06, 0.61)	−1.71 (1.68), *p* = .16	*t*_23_ = −1.48 (0.66), *p* = .01
	COMP	−2.61 (1.36), *p* = .03	0.37 (−0.01, 0.74)		Waitlist: *t*_23_ = −0.04 (0.92), *p* = .48 Treatment: *t*_23_ = −2.67 (1.0069), *p* = .01
Agoraphobia	ITT	−1.28 (0.65), *p* = .03	0.17 (0.00, 0.35)		Waitlist: *t*_23_ = −0.12 (0.46), *p* = .40 Treatment: *t*_23_ = −1.43 (0.46), *p* = .002
	COMP	−1.32 (0.68), *p* = .03	0.20 (0.00, 0.40)		Waitlist: *t*_23_ = −0.05 (0.50), *p* = .46 Treatment: *t*_23_ = −1.38 (0.47), *p* = .003
FQ	ITT	−4.43 (2.87), *p* = .06	0.19 (−0.05, 0.44)	−6.76 (5.44), *p* = .11	*t*_23_ = −2.95 (1.46), *p* = .02
	COMP	−5.14 (3.15), *p* = .053	0.23 (−0.05, 0.51)	−13.76 (5.50), *p* = .01	*t*_23_ = −2.54 (1.61), *p* = .06
BDI-II	ITT	−3.09 (2.00), *p* = .06	0.26 (−0.07, 0.60)	−1.62 (2.46), *p* = .26	*t*_23_ = −2.51 (1.02), *p* = .01
	COMP	−3.52 (1.94), *p* = .04	0.31 (−0.03, 0.64)		Waitlist: *t*_23_ = −0.53 (1.32), *p* = .35 Treatment: *t*_23_ = −3.99 (1.37), *p* = .003
Quality of life	ITT	−0.18 (2.05), *p* = .46	0.01 (−0.28, 0.25)	3.70 (3.45), *p* = .14	*t*_23_ = 0.61 (1.03), *p* = .28
	COMP	−0.14 (2.17), *p* = .47	0.01 (−0.28, 0.26)	6.67 (3.82), *p* = .04	*t*_23_ = −0.03 (1.09), *p* = .49

ITT, intention-to-treat; COMP, completers; ACS-30, Autonomy-Connectedness Scale-30; SA, Self-awareness; SO, Sensitivity to others; CMNS, Capacity for managing new situations; SCL-90, Symptom Checklist-90; FQ, Fear Questionnaire; BDI-II, Beck Depression Inventory II; QOL, Quality of life.

^†^Because the main effect of Time was different for t_2_ and t_3_, separate analyses were performed for t_2_ and t_3_.

#### Intention-to-Treat Analyses

**Table 4** shows that only on the SCL-90 Agoraphobia subscale was a significant (one-tailed) Time × Group interaction found, but not on any of the subscales of the ACS-30, the SCL-90 Anxiety subscale and total score, the FQ, BDI-II, and Quality of life. In the experimental treatment condition, contrasting the results for waitlist, agoraphobic symptoms decreased significantly. Effect sizes of the Time × Group interaction were generally negligible to small. As demonstrated by the significant Time effect, both groups improved with regard to Self-awareness, the SCL total score and Anxiety subscale, the FQ, and BDI-II, but not on the other scales (ACS-30 Sensitivity to other, ACS-30 Capacity for managing new situations, and Quality of life).

#### Completers Analyses

Results of the completers analyses (**Table 4**) demonstrated a significant (one-tailed) Time × Group interaction for Sensitivity to others, Capacity for managing new situations, the SCL total score, and the Anxiety and Agoraphobia subscales, as well as the BDI-II, but not on the other scales (ACS-30 Self-awareness, FQ, and Quality of life). Simple effects analyses showed that the experimental treatment condition improved with regard to Capacity for managing new situations, the SCL (total score as well as Anxiety and Agoraphobia subscales), and the BDI-II, whereas the waitlist condition did not show any significant change. Again, effect sizes of the Time × Group interaction were negligible to small. Additionally, the Time effect for Self-awareness was significant; both groups improved. Furthermore, the experimental treatment condition showed less agoraphobic symptoms (FQ) and a higher Quality of life than the waitlist condition as shown by the significant main effect of Group.

## Discussion

The present study examined the preliminary efficacy of a 15-session, group-based AET in a sample of patients with anxiety disorders. We compared AET with a waitlist control condition and expected AET to increase autonomy-connectedness and quality of life, as well as to reduce anxiety and comorbid depressive symptoms.

Results of the present study showed no indications of an increase of autonomy-connectedness and quality of life as a result of AET treatment. Also, AET did not seem to be able to alleviate depressive comorbidity. Nevertheless, the intention-to-treat and completers analyses both showed a larger decrease of agoraphobic symptoms in the experimental treatment than in the waitlist condition.

The reason the analyses showed the most consistent effect on agoraphobic symptoms may be explained by the fact that agoraphobia represented the largest diagnostic group in the present study’s sample (see **Table 1**). As a result, agoraphobic fears were likely often discussed during treatment. Also, next to direct effects of the treatment, attending a group-based therapy means patients have to expose themselves to their agoraphobic fears (e.g., leaving their home, using public transportation, and sitting in a small enclosed room with strangers).

Completers analyses, representing 75% of patients (*N* = 62) who completed treatment, showed additional effects on all anxiety-related measures assessed, as well as on depressive symptoms, often comorbid with anxiety. Completers analyses also showed an increase in capacity for managing new situations. This increase is in line with the beneficial effect on agoraphobic symptoms. That is, capacity for managing new situations reflects the decrease in agoraphobic symptoms, as patients become more flexible when entering new and feared situations.

As effect sizes were small with limited statistical power, and AET was tested in a preliminary pilot trial, we chose not to correct for multiple testing. Thereby, the overall Type I error rate exceeds 0.05, and results should be interpreted as preliminary and with caution. As all statistically significant results corresponded to *p*-values just below.05, all confidence intervals for the effect sizes subsequently contained zero. Thus, had we applied a correction for multiple testing, we would, strictly speaking, have concluded we found no evidence of an effect of AET. We, therefore, recommend more powerful future studies, that is, with sample sizes powerful enough to detect medium or even small population effect sizes (e.g., 200 persons per group gives a power of.8 to detect an effect size of *d* = .25), to find out if the present studies’ positive findings are mere chance findings or reflect true positive effects of the AET treatment on, for example, agoraphobic symptoms, and to uncover possible small–medium effects that we were unable to detect in the present study.

The first reason for the small effect sizes might be that the current sample suffered from quite severe psychopathology. Although severe symptoms leave much room for change, most patients (73.2%) received (multiple) previous treatment(s) for their anxiety symptoms but were unsuccessful so far, which may indicate a relatively high degree of treatment resistance in our sample. This type of patients is usual in the treatment centers that participated in our study, however. In addition, therapists indicated that group members with the most severe problems sometimes negatively affected the group process. For example, patients with additional cluster B personality problems are likely to draw more attention than the average group member and can disturb the group and impede treatment progression. Future research may, therefore, consider using more stringent inclusion criteria with regard to severity of certain comorbid disorders and ability to participate in a group treatment or providing training to therapists in the management of these types of patients if included.

Second, while patients are usually fully aware that they experience anxiety-related symptoms, they first have to receive psycho-education regarding their autonomy-related problems before fully understanding them. This may possibly lead to over-endorsement in their pre-treatment self-reports on the ACS-30, implying relatively small autonomy-connectedness difference scores between pre-treatment and post-treatment. Future research might, therefore, consider adding a retrospective pretest [e.g., Ref. (40)], allowing the measurement of response shifts (41), aside from clinical changes.

### Limitations

A limitation of the present study was that participants in the treatment condition had to wait up to a maximum of 6 weeks after the baseline assessment before treatment started, because we had to wait until we recruited enough patients before being able to start treatment. To avoid potential biases with regard to assessment intervals and study duration, we recommend equal moments of assessment of both groups in future studies.

Second, the consent rate was relatively low with 66%. Please note, however, that patients who did not consent did not necessarily deny AET treatment. As this trial also included a waitlist control condition, many patients may rather have been unwilling to wait 15 weeks to start their treatment.

Additionally, our patient sample was predominantly female, which means our results do not necessarily translate to males with an anxiety disorder. Other limitations include the use of self-report measures and the lack of follow-up measurements. Self-report measures are subject to social desirability [e.g., Ref. (42)]. Additionally, as long-term effects of AET are unknown, and effects may need some time to unfold, future research should consider adding follow-up assessments.

### Conclusion

Results should be interpreted in the context of the present study’s limitations. Although we found no effect of AET on autonomy-connectedness, quality of life, and depressive comorbidity, our results do tentatively suggest that this relatively short 15-session treatment may alleviate agoraphobic symptoms in a patient sample with severe anxiety. Here, it must be noted that the effects of AET in the present study were small and that we did not correct for multiple testing because of statistical power considerations. Future research with more stringent inclusion criteria is needed to further elucidate the effectiveness of AET. In the final session where treatment and treatment goals are evaluated, several patients explicitly stated *via* spontaneous comment that they evaluated AET as a non-aversive treatment when compared with earlier treatments they received. One of the reasons was that AET does not include explicit in-session exposure exercises. Feasibility, aversiveness, and transdiagnostic utility are important aspects to consider when evaluating treatments (43). AET requires further testing to determine whether it may serve as a promising alternative or addition to existing approaches.

## Data Availability

The raw data supporting the conclusions of the present paper will be made available by the authors, without undue reservation, to any qualified researcher and are stored in the Dataverse repository.

## Ethics Statement

The trial was approved by the medical ethics committee of the VU-University Medical Center and was carried out in compliance with the Code of Ethics of the World Medical Association (Declaration of Helsinki).

## Author Contributions

TB, MA, LR, and MB contributed to conception and design of the study. TB, MA, and MB were responsible for funding acquisition, supervision, and providing resources. LR, DJ, and MM were responsible for data collection, and LR was responsible for project administration. JM organized the data, and JM and MA performed the statistical analyses. JM wrote the first draft of the manuscript. All authors contributed to manuscript revision and read and approved the submitted version.

## Funding

This work was supported by VSB Fonds and Stichting tot Steun, VCVGZ.

## Conflict of Interest Statement

The authors declare that the research was conducted in the absence of any commercial or financial relationships that could be construed as a potential conflict of interest.
